# Non-monotonic kilohertz frequency neural block thresholds arise from amplitude- and frequency-dependent charge imbalance

**DOI:** 10.1038/s41598-021-84503-3

**Published:** 2021-03-03

**Authors:** Edgar Peña, Nicole A. Pelot, Warren M. Grill

**Affiliations:** 1 Department of Biomedical Engineering, Duke University, Room 1427, Fitzpatrick CIEMAS, 101 Science Drive, Campus Box 90281, Durham, NC 27708 USA; 2 Department of Electrical and Computer Engineering, Duke University, Durham, NC USA; 3 Department of Neurobiology, Duke University School of Medicine, Durham, NC USA; 4 Department of Neurosurgery, Duke University School of Medicine, Durham, NC USA

**Keywords:** Biophysical models, Peripheral nervous system, Biomedical engineering

## Abstract

Reversible block of nerve conduction using kilohertz frequency electrical signals has substantial potential for treatment of disease. However, the ability to block nerve fibers selectively is limited by poor understanding of the relationship between waveform parameters and the nerve fibers that are blocked. Previous in vivo studies reported non-monotonic relationships between block signal frequency and block threshold, suggesting the potential for fiber-selective block. However, the mechanisms of non-monotonic block thresholds were unclear, and these findings were not replicated in a subsequent in vivo study. We used high-fidelity computational models and in vivo experiments in anesthetized rats to show that non-monotonic threshold-frequency relationships do occur, that they result from amplitude- and frequency-dependent charge imbalances that cause a shift between kilohertz frequency and direct current block regimes, and that these relationships can differ across fiber diameters such that smaller fibers can be blocked at lower thresholds than larger fibers. These results reconcile previous contradictory studies, clarify the mechanisms of interaction between kilohertz frequency and direct current block, and demonstrate the potential for selective block of small fiber diameters.

## Introduction

Implanted neural stimulation devices for the treatment of disease are widespread and typically deliver electrical signals at tens to hundreds of hertz to evoke neural activity^1,2^. Less widely used are kilohertz frequency (KHF) waveforms that can block conduction of neural activity^3–5^. KHF signals produce persistent mean depolarization of the axonal membrane near the electrode contacts, causing sodium channel inactivation and local conduction block^6,7^. Preclinical studies of KHF nerve block for a wide range of disorders including diabetes^8^, heart failure^9^, and bladder control^10^ reflect the potential of this emerging technology. However, the relationship between waveform parameters and the nerve fibers that are blocked is poorly understood and this limits the ability to block selectively targeted nerve fibers.

Although most studies of KHF block report that the minimum current amplitude to achieve block increases with signal frequency^11–16^, Patel and Butera^17^ showed a non-monotonic effect of signal frequency on block threshold. In experiments on rat vagus and sciatic nerves, using sinusoidal KHF signals, frequencies ≤ 30 kHz blocked faster conducting fibers at lower thresholds, while frequencies ≥ 50 kHz blocked more slowly conducting fibers at lower thresholds; this raises the important possibility of fiber-type selective block by choosing an appropriate signal frequency. However, these findings were not replicated in a subsequent study in which both slow and fast conducting fibers of the rat vagus nerve exhibited monotonically increasing block thresholds with frequency, and the slow fibers had higher block thresholds at all frequencies^18^. Non-monotonic frequency effects are unexpected because the passive properties of the axonal membrane attenuate high frequencies irrespective of fiber diameter or myelination, and this attenuation underlies the increase in block thresholds at higher frequencies^19^. The non-monotonic thresholds in the former study^17^ may be due to unintended charge imbalances in the waveforms generated by the instrumentation^20^ which modulated the threshold-frequency relationships; this explanation is consistent with computational modeling studies of charge-imbalanced asymmetric waveforms which also produced non-monotonic block thresholds^21,22^. However, those modeling results did not clarify the relative roles of charge imbalance and waveform asymmetry in determining block thresholds, and the lack of experimental data limits the relevance to in vivo applications. In vivo data are particularly crucial given the potential of direct current (DC) to damage nerves^23^, potentially limiting long-term use of this technique.

We conducted a comprehensive study to quantify the effects of charge imbalance, frequency, and asymmetry of KHF signals on block thresholds using computational models and in vivo experiments. We characterized the interactions between the KHF and DC contributions to conduction block to reveal how frequency-dependent thresholds emerge from waveform characteristics. The results demonstrate that amplitude- and frequency-dependent charge imbalance resulted in non-monotonic block thresholds across frequencies, such that block was generated by the KHF component at low frequencies and by the DC component at high frequencies. The interactions between KHF and DC effects resulted in instances of block that were selective for smaller diameter model nerve fibers, and these interactions produced complex, polarity-dependent effects on block, transmission, and excitation across frequencies and KHF amplitudes. Our data provide the first experimental evidence of non-monotonic effects of frequency with charge-imbalanced waveforms, harmonize previous contradictory findings, and clarify the mechanisms of interaction between KHF and DC that can be leveraged for fiber-selective block.

## Methods

Using a computational model of the rat tibial nerve and in vivo recordings of rat gastrocnemius muscle force, we quantified the effects of charge imbalance, frequency, and asymmetry of KHF signals on block thresholds across a suite of biphasic rectangular KHF waveforms mixed with different levels of DC. We conducted all data analyses and statistics in MATLAB R2018a (Mathworks; Natick, MA).

### Nerve block waveforms

**Figure 1:**
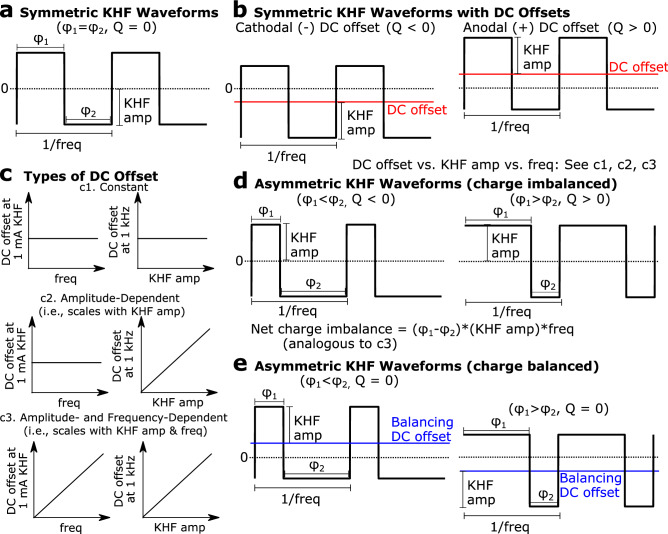
Waveforms tested to independently analyze blocking effects of DC offset types, asymmetric charge imbalance, and asymmetry. KHF amplitude was defined as half of the peak-to-peak amplitude of each waveform. (**a**) Symmetric KHF waveform with zero net charge per unit time (Q = 0). (**b**) Symmetric KHF waveforms with added DC offsets, where symmetry was defined as equal duration phases. Net charge per unit time (Q) was either negative (cathodal DC offset) or positive (anodal DC offset). (**c**) Types of DC offset added to symmetric KHF waveforms. DC offsets were either constant (i.e., independent of KHF parameters) (c1), amplitude-dependent (i.e., scaled with KHF amplitude only) (c2), or amplitude- and frequency-dependent (i.e., scaled with both KHF amplitude and frequency) (c3). (**d**) Asymmetric KHF rectangular waveforms were constructed from the symmetric waveform by defining unequal phase durations. Differences in phase durations (± 2, 3, 4 μs) were independent of waveform frequency, such that the net charge per unit time scaled linearly with KHF amplitude and with frequency, analogous to the DC offset type illustrated in (c3). (**e**) Asymmetric waveforms with a compensatory DC offset that produced zero net charge per unit time.

We evaluated a suite of rectangular waveforms in computational models and in vivo (1) to identify the properties of nerve block instrumentation that could lead to non-monotonic block thresholds, and (2) to probe the mechanisms of non-monotonic block thresholds by disentangling the individual contributions of waveform components to block thresholds across frequencies. In computational models, we probed the type of DC offset necessary for non-monotonic block thresholds by comparing *symmetric* rectangular waveforms with zero net charge (Fig. 1a) against symmetric rectangular waveforms with added or subtracted DC offsets (Fig. 1b), where “symmetry” refers to equal duration phases. We evaluated three different types of DC offset, corresponding to hypothetical nerve block instruments with distinct dependencies between a KHF signal and unintended DC offsets (Fig. 1c, subpanels c1, c2, c3): (1) “constant DC offset” that was independent of any KHF parameter; (2) “amplitude-dependent DC offset” that scaled linearly with KHF amplitude; (3) “amplitude- and frequency-dependent DC offset” that scaled linearly with both KHF amplitude and frequency. KHF amplitude was defined as half of the peak-to-peak amplitude in all cases (Fig. 1b). Constant DC offset values were ± 15, ± 26, ± 46, ± 80, ± 106, ± 141, ± 186, ± 246, ± 326, ± 431, ± 754, and ± 1,320 μA. Amplitude-dependent DC offset values were ± 10, ± 20, ± 40, ± 59, ± 77, ± 100, ± 125, ± 143, ± 167, ± 200, and ± 400 μA per mA of KHF. Amplitude- and frequency-dependent DC offset values were ± 0.5, ± 1, ± 1.5, ± 2, ± 2.5, ± 3, ± 3.5, and ± 4 μA per mA of KHF per 1 kHz. The choice of DC offsets was based on preliminary simulations, and spanned the relevant range of values such that the smallest offsets had little or no effect while the largest offsets had a saturated or nearly saturated effect. We evaluated all waveforms at 10, 20, 29.4, 38.5, 50, 62.5, 71.4, 83.3, and 100 kHz. These frequencies had periods that were integer multiples of 1 μs to ensure that waveform discretization in computational models resulted only in the intended amounts of charge imbalance.

Previous computational modeling studies^22^ evaluated block thresholds of asymmetric rectangular waveforms, corresponding to hypothetical nerve block instruments that generate waveforms with unintended asymmetry. While such waveforms produced non-monotonic block thresholds, the individual contributions of asymmetry and charge imbalance were unclear. Therefore, we evaluated two types of *asymmetric* waveforms that—along with our tests of symmetric waveforms with DC offsets—enabled analysis of individual contributions of asymmetry and charge imbalance to non-monotonic block thresholds. The first type of asymmetric waveform replicated the asymmetry from the previous study (Fig. 1d), such that the differences in duration between the first and second phases (in μs) were constant across all frequencies and thus produced net charge per unit time (Q), i.e., DC, that scaled with KHF amplitude and frequency, similar to that illustrated in Fig. 1c, subpanel c3. A phase difference of 1 μs produced equivalent net charge per unit time (Q) as that produced by an amplitude- and frequency-dependent DC offset of 1 μA DC per mA KHF per 1 kHz. The second type of asymmetric waveform was constructed from the first type with a compensatory DC offset that resulted in zero net charge per unit time (Fig. 1e). We simulated computational models at the same frequencies as the symmetric waveforms described above to evaluate block thresholds for both types of asymmetric waveforms with phase differences of ± 2, ± 3, and ± 4 μs.

We conducted in vivo experiments to validate the predictions from computational models of symmetric waveforms without DC offsets (Fig. 1a), symmetric waveforms with DC offsets (Fig. 1b) that were amplitude- and frequency-dependent (Fig. 1c, subpanel c3), and asymmetric waveforms that were charge-imbalanced (Fig. 1d) and charge-balanced (Fig. 1e). The symmetric waveforms with DC offsets were offset by ± 2, ± 3, ± 4 μA DC per mA KHF per 1 kHz. Phase differences for asymmetric waveforms were ± 2, ± 3, and ± 4 μs, enabling direct comparison between DC offset symmetric waveforms and charge-imbalanced asymmetric waveforms. The phase differences, in turn, were in a range similar to previous modeling work on asymmetric charge-imbalanced waveforms^22^, facilitating comparisons of the present symmetric and asymmetric work to previous studies. We evaluated all waveforms in vivo at 20, 40, 60, and 80 kHz.

**Figure 2:**
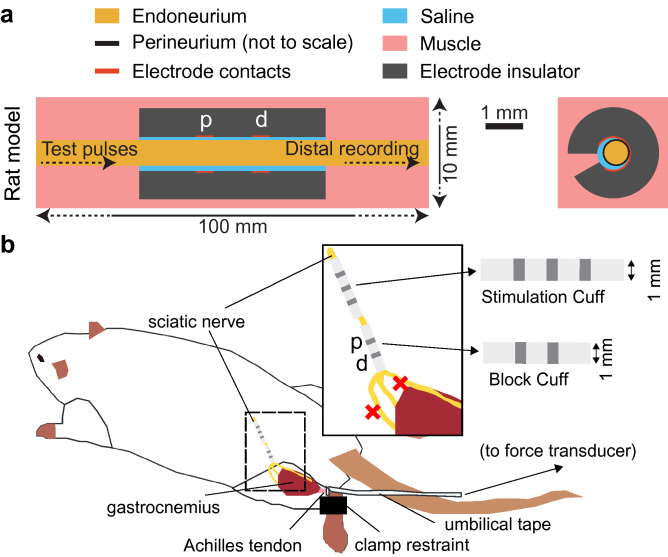
Finite element model of rat tibial nerve with bipolar cuff electrode (**a**), and analogous in vivo experimental setup targeting rat tibial nerve (**b**). The “p” and “d” labels indicate the proximal and distal contacts, respectively. In the computational model in panel (**a**), test pulses were evoked near the proximal end and the transmembrane potential was recorded near the distal end of each axon modeled within the endoneurium. In the in vivo setup in panel (**b**), the cuff electrodes were placed on the sciatic nerve; the common peroneal and sural branches were transected (red X’s), as well as the branches innervating the hamstring (not shown), and signals were transmitted to the gastrocnemius via the tibial branch.

All waveforms were evaluated at positive and negative polarities, corresponding to positive or negative DC offsets or phase differences (Fig. 1b,d,e). Unless otherwise specified, we refer to polarity in terms of the proximal contact of the bipolar blocking electrode, such that negative (or cathodal) DC and positive (or anodal) DC correspond to current sinks and current sources at the proximal electrode contact, respectively (see Fig. 2 and corresponding Methods text for electrode orientation details).

### Computational models

#### Finite element models of rat tibial nerve

We implemented a finite element model (FEM) of a rat tibial nerve and cuff electrode using COMSOL Multiphysics v5.3a (Burlington, MA) (Fig. 2a). The monofascicular rat tibial nerve was modeled as a 0.75 mm diameter cylinder^24,25^ surrounded by a bipolar cuff electrode (contacts 0.5 mm in length spaced 1 mm edge-to-edge; 1.5 mm between each edge of the cuff and the nearest contact edge; 5 mm total cuff length; 0.875 mm insulator thickness; 1 mm inner diameter); the insulator surrounded 330° of the nerve circumferentially and the contacts spanned 270°. We positioned the nerve 10 μm away from the inner wall of the cuff that was opposite the cuff opening, and the cuff was centered along the length of the 100 mm-long nerve. We placed a point current source within each of the platinum ribbon electrode domains (+ 1 mA in the proximal contact and − 1 mA in the distal contact), in accordance with a methods study on modeling current sources for neural stimulation in COMSOL^26^. We grounded all outermost surfaces of the model except the ends of the nerve. We modeled the insulator of the cuff as silicone (1e12 Ω-m^27^) and the contacts as platinum (1.06e−7 Ω-m^28^). We modeled the endoneurium as an anisotropic medium (1.75 Ω-m longitudinally, 6 Ω-m radially^29,30^), the perineurium using a thin layer approximation (COMSOL’s contact impedance boundary condition; thickness equal to 3% of the fascicle diameter^31^; 1149 Ω-m^30^), the space between the nerve and the cuff as isotropic saline (0.568 Ω-m^32^), and the rest of the tissue outside the nerve and cuff as anisotropic muscle (2.86 Ω-m longitudinally, 11.6 Ω-m radially^33^; 10 mm diameter).

We meshed the 100 mm-long FEM with 1,510,090 tetrahedral elements. We used quadratic geometry and solution shape functions, and the conjugate gradients solver to solve Laplace’s equation for potentials in the volume assuming quasi-static conditions and non-dispersive materials^34^. We doubled the mesh density until the block threshold for a 10 kHz symmetric rectangular wave with zero offset applied to a 100 mm-long, 5.7 μm diameter axon at the center of the nerve changed < 3%^35^.

#### Simulations of biophysical axons

We applied the electric potentials from the FEM to 100 mm-long model axons centered in the nerve. We simulated mammalian myelinated axons using the McIntyre-Richardson-Grill (MRG) model^36,37^ in NEURON v7.5^38^. We used 5.7 μm-diameter axons for most simulations and 5.7, 7.3, 8.7, 10, and 11.5 μm-diameter axons for the comparisons of effects across fiber diameters. The chosen range of fiber diameters is representative of those reported for rat tibial nerve^24^. We included passive end nodes to reduce edge effects (g_m_ = 0.0001 S/cm^2^, c_m_ = 2 μF/cm^2^, − 70 mV reversal potential). The middle node of Ranvier of each axon was aligned with the middle of the FEM.

We initialized each simulation with 10 ms time steps from t = − 200 ms to t = 0 ms to ensure initial steady-state and ran each simulation from t = 0 ms to t = 250 ms with 0.5 µs time steps (backward Euler integration). We delivered supra-threshold 2 nA intracellular test pulses every 50 ms starting at t = 25 ms at the node of Ranvier closest to 6 mm from the proximal end of the nerve. We delivered the KHF waveform starting at t = 1 ms. For each KHF waveform, we scaled the potentials obtained from the FEM to simulate amplitudes from 0.05 to 5 mA in 6% increments. We counted the action potentials at the node of Ranvier closest to 12 mm from the distal end of the nerve starting at t = 100 ms, which allowed sufficient time for the onset response to subside^15^. “Transmission”, “block”, and “excitation” were defined in terms of recorded action potentials between 100 and 250 ms. “Transmission” was the presence of exactly three action potentials spaced 50 ms apart (1 ms tolerance) in response to the test pulses at t = 125, 175, and 225 ms, with the first action potential occurring within 5 ms of a test pulse (i.e., allowing for conduction delay). “Block” was the total absence of action potentials after t = 100 ms. “Excitation” was anything that was neither “transmission” nor “block”. “Block threshold” was the minimum amplitude that produced block. To prevent spurious block threshold measurements in computational models, we further required that block be maintained at least 0.1 mA above block threshold, except in two simulations with block windows that were truly smaller than 0.1 mA (i.e., symmetric rectangular waves at 10 kHz with + 167 and + 200 μA DC offset per mA KHF).

#### In vivo electrical block of the rat tibial nerve

We conducted acute experiments to quantify in vivo responses of the tibial nerve to KHF signals in male Sprague–Dawley rats (n = 7; 362 to 678 g, median = 440 g; Charles River Laboratories) by recording the force generated by the gastrocnemius (Fig. 2b). All procedures were approved by the Institute for Animal Care and Use Committee of Duke University (Durham, NC) and were in accordance with the Guide for Care and Use of Laboratory Animals (8th edition). The study was also carried out in compliance with the ARRIVE guidelines^41^. The animals were housed under USDA- and AAALAC-compliant conditions, with 12 h/12 h light/dark cycle and free access to food, water, and environmental enrichment. Rats were placed in an anesthesia box, briefly anesthetized with 3% isoflurane in air, and then injected subcutaneously with 1.2 g/kg urethane, with supplemental doses administered as required (up to 0.4 g/kg total; SQ, IM, or IP). Heart rate and blood oxygenation were monitored continuously using a pulse oximeter (PalmSAT 2500A; Nonin Medical; Plymouth, MN, USA), and depth of anesthesia was assessed using the toe pinch reflex and heart rate. Body temperature was monitored using a rectal temperature probe (TH-8 Thermalert; Physitemp Instruments, Inc.; Clifton, NJ) and maintained between ~ 35–38 °C with a heated water blanket.

We adapted the surgical methods described in a prior publication^11^ to measure the effects of KHF signals on the rat tibial nerve in vivo. An incision was made on the left hind limb from the distal dorsal ankle to 1 cm rostral to the ipsilateral hip joint. The muscle overlying the gastrocnemius was cut parallel to the skin incision to expose the gastrocnemius and the sciatic nerve. The connective tissue surrounding the sciatic nerve was dissected from ~ 0.5 cm caudal to the spinal cord to the branching point into the tibial, common peroneal, and sural nerves. The common peroneal and sural nerves were transected, as well as the branches of the sciatic nerve innervating the hamstring, leaving only the tibial branch intact. The gastrocnemius was dissected from the tibia. The Achilles tendon was dissected and cut at its distal end, and the tendon was tied to a custom strain gauge-based force transducer using umbilical tape. The tibia was secured at its caudal end by a plastic clamp that was attached to the experimental table.

We placed a tripolar cuff on the proximal sciatic nerve to deliver test pulses to contract the gastrocnemius and a bipolar cuff on the distal sciatic nerve to deliver the KHF waveforms. The tripolar cuff (1 mm inner diameter; X-Wide Contact Cuffs, Microprobes; Gaithersburg, MD) contained three Pt-Ir 90–10 ribbon contacts (0.5 mm wide) spaced 1 mm apart edge-to-edge; the cuff was 6.5 mm in length total, including 1.5 mm of silicone beyond the outer edge of each outer contact. The bipolar cuff (1 mm inner diameter; X-Wide Contact Cuffs, Microprobes; Gaithersburg, MD) contained two Pt-Ir 90–10 ribbon contacts (0.5 mm wide) spaced 1 mm apart edge-to-edge; the cuff was 5 mm in length total, including 1.5 mm of silicone on each end. The silicone thickness of both cuffs was 0.875 mm. After implanting the cuffs at the start of each experiment, we measured the impedance between the middle contact and the shorted outer contacts of the tripolar cuff (impedance at 10 kHz: 0.82 to 1.30 kΩ; median = 0.92 kΩ) and between the contacts of the bipolar cuff (impedance at 10 kHz: 2.00 to 3.20 kΩ; median = 2.70 kΩ). After placement, the two cuffs were spaced ~ 0.2 to 0.5 cm edge-to-edge.

Stimulation signals and recorded muscle force were controlled and sampled by a computer and PowerLab/4SP (ADInstruments Inc.; Colorado Springs, CO). Custom MATLAB scripts controlled and synchronized all stimulation and recording protocols. The signals from the force transducer were amplified at 10x (ETH-255; CB Sciences Inc.; Dover, NH) and were digitized and recorded by the PowerLab unit interfaced via LabChart v7.0 (f_s_ = 200 samples/s, 50 Hz digital low pass filter; ADInstruments). Voltage signals from the PowerLab unit drove a voltage-to-current stimulus isolator (A-M Systems 2200; Sequim, WA) to deliver biphasic symmetric test pulses (0.2 ms/phase) to the tripolar cuff (cathodal phase first to the middle contact and anodal phase first to the shorted outer contacts) via a DC offset removal circuit (100 kΩ resistor in parallel with the stimulus isolator and a 1 μF capacitor in series with the isolator output; based on a previous study^20^). The test pulses had higher amplitudes than required to generate maximal twitches of the gastrocnemius muscle (~ 0.7 to 1 mA). A voltage-to-current high power stimulus isolator with 1 MHz bandwidth (A-M Systems 4100) delivered KHF waveforms to the bipolar cuff with the positive output connected to the proximal contact such that “cathodal” or “anodal” stimulation from the computational models matched “cathodal” or “anodal” stimulation from experiments. The KHF signals were generated by a computer-controlled current source (Keithley 6221; Tektronix, Inc; Beaverton, OR) that was triggered by MATLAB through a National Instruments VISA connection; the output of the Keithley was passed through a 100 Ω resistor and the voltage across this resistor was supplied as input to the A-M Systems 4100 on the 10 × input gain setting. We did not include a DC offset removal circuit between the KHF signal source and the cuff electrode because an explicit goal of our study was to evaluate the effects of charge imbalances. Rather, prior to every experiment, we calibrated the A-M Systems 4100 such that shunting its inputs produced less than 2 μA DC offset current at the output across a 1 kΩ resistor. In addition, we monitored the KHF signal during the experiments by visualizing the voltage across a 100 Ω resistor in series with the bipolar cuff using a battery-powered oscilloscope (Fluke 190–062 ScopeMeter Test Tool; Fluke Corporation; Everett, WA).

We measured block threshold (i.e., the minimum current required to produce nerve block) for each waveform-frequency pair using a low-to-high search followed by a binary search. We randomized the order of all waveforms to be tested and then randomized the order of the four frequencies for each waveform (20 to 80 kHz, Δ = 20 kHz). During each test, we applied a KHF signal at an initial amplitude between 1 to 3.5 mA (charge-balanced waveforms) or between 0.2 to 0.5 mA (charge-imbalanced waveforms). We increased the amplitude if the initial amplitude did not block and repeated this process until we identified a supra-block amplitude. We then conducted a standard binary search by iteratively applying the mean of the largest non-blocking amplitude and the smallest blocking amplitude until we observed a difference between the search bounds of less than 0.2 mA (charge-balanced waveforms) or 0.1 mA (charge-imbalanced waveforms). We applied the test pulses at 1 Hz, except for the charge-imbalanced waveforms tests at 80 kHz, where we used 2 Hz due to the short duration of those tests (see below). We determined the presence or absence of nerve block visually based on the presence or absence of gastrocnemius contraction in force recordings displayed in real-time in LabChart.

We employed three strategies to reduce the application of non-zero net charge and therefore reduce the risk of permanent impairment of nerve conduction^23^. We set our initial KHF amplitudes to be markedly lower for charge-imbalanced waveforms, as stated above, and the duration of each delivery of a KHF signal was short: 2 s (80 kHz), 3 s (60 kHz), 4 s (40 kHz), or 5 s (20 kHz) for the charge-imbalanced waveforms and 5 s for all charge-balanced waveforms. Further, for a given waveform, frequency, and amplitude, we evaluated both polarities (i.e., cathodal and anodal) consecutively (with 2 s pause in between) to achieve zero net charge over each pair of tests. We paused for > 2 s between amplitudes and > 5 s between each waveform and frequency pair. In addition to expediting the experiment, the short duration signals and low initial amplitudes also reduced the possibility of confounding carryover effects^18,39,40^, which were not observed in this study. In nerves 1–3, we terminated each binary search after identifying the minimum amplitude that blocked nerve conduction regardless of polarity, taking the block threshold only of the polarity that blocked at a lower threshold. In nerves 4–7, we extended each threshold search to measure block threshold at both polarities consecutively when polarity effects were evident.

Rats were euthanized at the termination of experiments with Euthasol (0.5 ml IP; Virbac; Fort Worth, TX) and bilateral thoracotomy within 12 h of the initial urethane dose.

### Data analysis and statistics

We quantified the effects of DC offset on block thresholds measured in vivo using the following mathematical model:

1 $$T={T}_{0}{e}^{-m\left|L\right|f/({L}_{max}*{f}_{max})}$$

where T is the block threshold of a waveform with a DC offset, T_0_ is the block threshold of the same waveform without a DC offset, f is the frequency in kilohertz, L is the level of amplitude- and frequency-dependent DC offset in μA DC per mA KHF per 1 kHz, m is a coefficient to be fit, L_max_ is the maximum DC offset level evaluated in μA DC per mA KHF per 1 kHz, and f_max_ is the maximum frequency evaluated in kilohertz. In the presence of a non-zero DC offset, for the KHF signals with amplitude- and frequency-dependent DC offsets that we evaluated in vivo, Eq. 1 specifies that block threshold decays toward zero as DC offset or frequency increase. We further extended the mathematical model with three additional variables to account for the presence of two distinct DC offset polarities and for the fact that we obtained repeated measures of each nerve and each frequency:

2 $$T={p}_{i}{a}_{j}{c}_{k}{T}_{0}{e}^{-m\left|L\right|f/({L}_{max}*{f}_{max})}$$

Parameters p_i_, a_j_, and c_k_ were adjustment factors for a specific polarity i, a specific nerve j, and a specific frequency k, respectively. We set L_max_ to 4 μA DC per mA KHF per 1 kHz, set f_max_ to 80 kHz, and took the natural log of both sides of the Eq. 2 to produce the following linear equation:

3 $$\mathrm{ln}T =\mathrm{ln}{p}_{i}+\mathrm{ln}{a}_{j}+\mathrm{ln}{c}_{k}+\mathrm{ln}{T}_{0}-m*\frac{\left|L\right|f}{4*80}$$

We fit Eq. 3 to our in vivo data quantifying block for symmetric waveforms with DC offsets using a three-way ANCOVA with one covariate (anovan function in MATLAB R2018a, setting polarity, nerve index, and frequency as categorical grouping variables, and DC offset as a continuous variable). We also separately fit Eq. 3 to measurements of charge imbalance effects due to asymmetric waveforms. We verified approximate normality of residuals using Q-Q plots and residual histograms, and we report results of Anderson–Darling tests for normality.

## Results

We quantified the block thresholds for a suite of symmetric and asymmetric biphasic KHF waveforms (Fig. 1), including charge-balanced and -imbalanced waveforms, using both computational models and in vivo experiments (Fig. 2). We implemented a finite element model of the rat tibial nerve coupled to biophysically-realistic models of myelinated axons. We stimulated the rat tibial nerve in vivo and recorded the resulting gastrocnemius force.

### Non-monotonic block thresholds across frequencies are due to amplitude- and frequency-dependent charge imbalance

**Figure 3:**
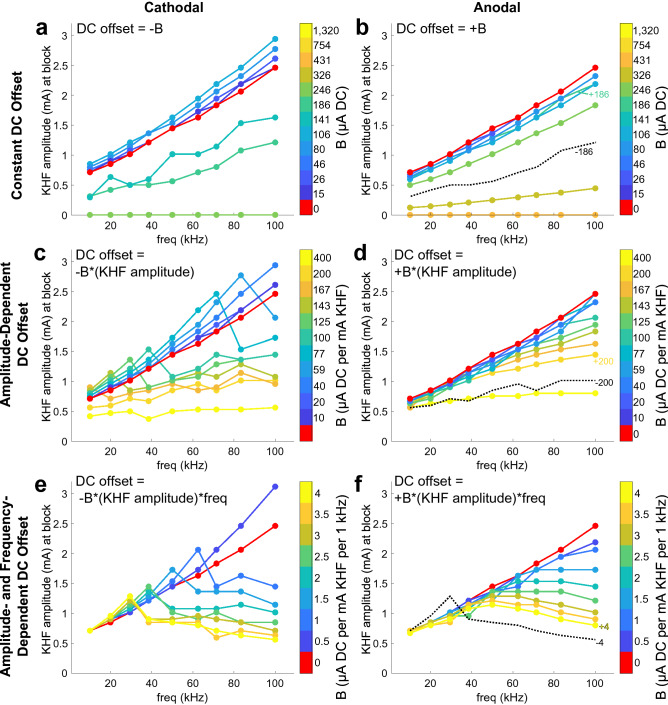
Frequency effects on block thresholds in computational models of symmetric KHF rectangular waves with different types of DC offsets. Polarities apply to the proximal contact of the bipolar cuff (Fig. 2) and the signs of the DC offsets are for the current on the proximal contact. Model axons were myelinated and had a 5.7 μm fiber diameter. KHF amplitude indicates half of the peak-to-peak amplitude of the KHF waveform. (**a**,**b**) Block thresholds due to constant DC offset for cathodal (**a**) and anodal (**b**) polarities. The data for the four (**a**) and three (**b**) highest levels of DC are overlaid at zero threshold. Thresholds at 1320 μA are not shown for cathodal DC because this amplitude produced only DC excitation. The black dotted line on the anodal DC plot shows the − 186 μA data from the cathodal DC plot. (**c**,**d**) Block thresholds of KHF waveforms with cathodal (**c**) and anodal (**d**) DC offsets that scale with KHF amplitude. The black dotted line on the anodal DC plot shows the − 200 μA per mA KHF data from the cathodal DC plot. (**e**,**f**) Block thresholds for KHF waveforms with cathodal (**e**) and anodal (**f**) DC offsets that scale with KHF amplitude and frequency. The black dotted line on the anodal DC plot shows the − 4 μA per mA KHF per 1 kHz data from the cathodal DC plot.

We first investigated block thresholds using symmetric rectangular waves with various DC offsets (Fig. 1a–c). The effects of DC offsets differed with the type (constant, amplitude-dependent, amplitude- and frequency-dependent; Fig. 1c), amount, and polarity of DC. Quantifying the effects of DC offsets on block thresholds in a computational model of 5.7 μm myelinated fibers from 10 to 100 kHz revealed that non-monotonic effects of frequency on block threshold resulted from amplitude- and frequency-dependent charge imbalances (Fig. 3).

Small amounts of constant DC had polarity-dependent effects on block thresholds, but in all cases, block thresholds increased with frequency for a given constant level of DC (Fig. 3a,b). Comparing across levels of DC, cathodal DC (i.e., net cathodal current on the proximal contact; Fig. 2) up to − 106 μA increased block thresholds for all frequencies (Fig. 3a), while anodal DC up to + 246 μA decreased block thresholds for all frequencies (Fig. 3b). Block thresholds dropped abruptly at higher levels of constant cathodal (beyond − 141 μA) and anodal (beyond + 326 μA) DC, reaching zero for both polarities by ± 431 μA; thresholds of zero corresponded to the DC component producing nerve block on its own, irrespective of KHF amplitude or frequency.

Cathodal DC offsets that scaled with KHF amplitude either increased block thresholds at a given frequency when frequencies were low, or decreased thresholds when frequencies were high (Fig. 3c, − 59 to − 167 μA per mA KHF). This transition happened at a particular ‘knee’ frequency that was inversely related to the magnitude of DC offset (i.e., parameter “B” in Fig. 3c). Below the knee frequency, the effects of cathodal DC were qualitatively similar to smaller amplitudes of constant cathodal DC (e.g., Fig. 3a, − 15 to − 141 μA). Above the knee frequency, the effects were similar to larger amplitudes of constant cathodal DC (e.g., Fig. 3a, − 186 μA). Importantly, block thresholds increased monotonically with frequency before and after the knee frequency. Anodal DC offsets (Fig. 3d) that scaled with KHF amplitude decreased block thresholds at any given frequency, similar to constant anodal DC offsets (Fig. 3b). Block thresholds for amplitude-dependent DC did not drop to zero because the DC amplitude was dependent on the KHF amplitude so DC block could not occur at zero. However, by 400 μA DC per mA KHF, the effects of frequency on block thresholds were substantially muted for both polarities.

DC offsets that scaled with both KHF amplitude and frequency (Fig. 3e,f) uniquely produced block thresholds that changed non-monotonically with frequency, first increasing and then decreasing as frequency was increased. Cathodal DC offsets that were dependent on both KHF amplitude and frequency exhibited a ‘knee’ frequency (Fig. 3e) similar to those of Fig. 3c, except that thresholds decreased with frequency after the ‘knee’ (Fig. 3e). Anodal DC offsets that were dependent on both KHF amplitude and frequency produced lower block thresholds with greater offset (Fig. 3f) similar to effects in Fig. 3d, except that thresholds increased then decreased with frequency at DC offset levels greater than or equal to 1.5 μA DC per mA KHF per 1 kHz for the range of frequencies examined.

**Figure 4:**
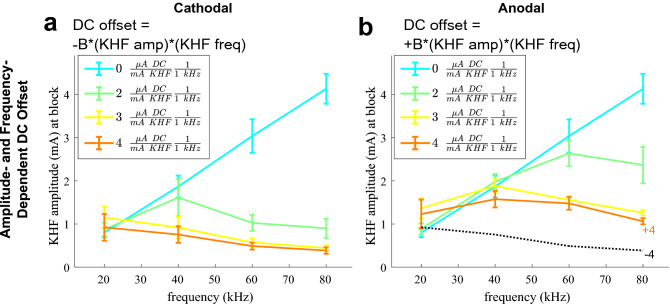
Frequency effects on block thresholds during in vivo rat tibial nerve experiments. Symmetric KHF rectangular waves were offset by cathodal (**a**) or anodal (**b**) DC that scaled with KHF amplitude and frequency. Plots show mean and standard error of the mean of block thresholds across three to seven nerves. The black dotted line on the anodal DC offset plot shows the − 4 μA per mA KHF per 1 kHz data from the cathodal DC offsets plot. See Supplemental Fig. 1 for individual nerve data points.

In vivo experiments confirmed the non-monotonic frequency effects of amplitude- and frequency-dependent DC offsets for symmetric waveforms (Fig. 4; Supplemental Fig. 1). In all rat tibial nerves tested, KHF signals with zero DC offset exhibited block thresholds that increased monotonically with frequency. Conversely, all waveforms with DC offsets that depended on both KHF amplitude and frequency exhibited block thresholds that varied non-monotonically with frequency. Waveforms with greater DC offset magnitude (i.e., parameter “B” in Fig. 4) generally exhibited lower block thresholds at a given frequency and a maximum threshold that occurred at a lower frequency. Equation 3 fits showed a linear relationship between the degree of DC offset (|L|*f) and the natural log of block thresholds (m = 2.3; CI = [2.0, 2.6]; adjusted R^2^ = 0.69; F(11,123) = 27.68; p-value = 3e−28), with minor deviations of residuals from normality (Anderson–Darling test p-value: 0.0263).

In computational models and in vivo experiments, cathodal DC offsets of a given level reduced block thresholds more than anodal DC offsets of the same level (examples marked in black dashed lines and corresponding labeled colored lines in Figs. 3b,d,f and 4b). Exceptions occurred in computational models when cathodal DC offsets were small enough to increase block thresholds (e.g., below knee frequency), although this phenomenon was not consistent during in vivo experiments (Fig. 4b).

### Charge-imbalanced asymmetry but not charge-balanced asymmetry produced non-monotonic threshold-frequency relationships

**Figure 5:**
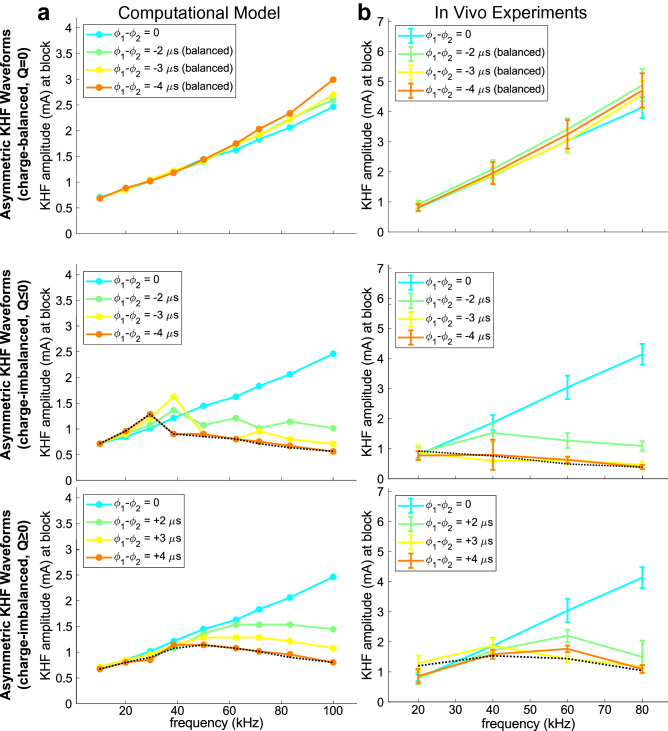
Frequency effects of asymmetric waveforms in computational models (**a**) and in vivo experiments (**b**). Waveforms were either charge-balanced with asymmetric phases plus compensatory DC offsets to cancel out imbalances (top row; Fig. 1e) or charge-imbalanced with asymmetric phases (middle and bottom rows; Fig. 1d). KHF amplitude was half of the peak-to-peak amplitude of the KHF waveform for all waveforms. The amount of asymmetry is shown as the difference in duration between the first (φ_1_) and second (φ_2_) phases of the biphasic KHF waveforms. The black dotted line in each charge-imbalanced waveform plot shows the corresponding ± 4 μA per mA KHF per 1 kHz line from Fig. 3 (computational model) or Fig. 4 (in vivo), which produced the same net charge per unit time as asymmetric charge-imbalanced waveforms with ± 4 μs phase difference. In charge-balanced asymmetric waveforms, only negative phase differences are shown, as the sign of asymmetry had no effect on threshold-frequency relationships. The 0 μs phase difference (cyan) lines for in vivo data are from the same data as in Fig. 4. See Supplemental Fig. 2 for individual nerve data points.

While the above sections examined charge-balanced and -imbalanced *symmetric* waveforms, we also examined the responses to *asymmetric* waveforms (Fig. 1d,e). In computational models (Fig. 5a) and in vivo experiments (Fig. 5b), block threshold increased monotonically with frequency for charge-balanced asymmetric waveforms, and asymmetry had little to no effect on peak-to-peak KHF amplitude at block threshold, with slight increases in block threshold due to asymmetry at ≥ 60 kHz in computational models. Conversely, we observed non-monotonic block threshold-frequency relationships with charge-imbalanced asymmetric waveforms. The effects of charge-imbalanced asymmetric waveforms were similar to the effects of symmetric waveforms that had an equivalent level of amplitude- and frequency-dependent DC offset (e.g., Fig. 5 black dashed lines vs. orange lines comparing ± 4 μs phase differences vs. ± 4 μA DC offset per mA KHF per 1 kHz, data from Figs. 3e,f and 4a,b). The trends observed were consistent across computational models and in vivo experiments. Equation 3 fits showed a linear relationship between the degree of net charge imbalance per unit time (|L|*f) and the natural log of block thresholds (m = 2.2; CI = [2.0, 2.5]; adjusted R^2^ = 0.65; F(11,132) = 24.93; p-value = 4e−27), with normal residuals (Anderson–Darling test p-value: 0.4413), and this was consistent with effects of DC offset in symmetric waveforms. Therefore, the non-monotonic effects of charge imbalance occurred irrespective of whether the charge imbalance was due to translational DC offsets or an equivalent amount of charge per unit time from unequal phase durations.

### Non-monotonic block thresholds transitioned from charge-balanced KHF thresholds at low frequencies to amplitude- and frequency-dependent DC thresholds at high frequencies

**Figure 6:**
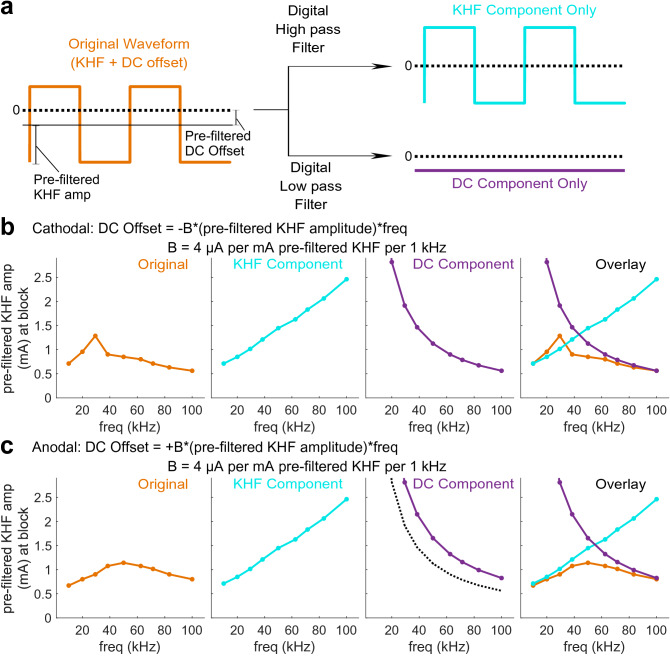
Computational models of block thresholds across frequencies for KHF and DC offset components separately in a 5.7 μm diameter fiber. (**a**) Original waveforms consisted of KHF symmetric rectangular waves with added DC offset that scaled with KHF amplitude and frequency. Digital high pass or low pass filters preserved only the KHF or DC components of the original signal, respectively. DC offsets were either cathodal (**b**) or anodal (**c**) at ± 4 μA DC per mA pre-filtered KHF amplitude per 1 kHz. (**b**,**c**) KHF amplitude of Original waveforms (y-axis) required for block with Original waveforms (orange), KHF component only (cyan), or DC component only (purple). Threshold curves for Original waveforms in (**b**,**c**) were identical to the corresponding ± 4 μA DC per mA KHF amplitude per 1 kHz curves in Fig. 3e,f. Threshold curves for KHF components in (**b**,**c**) were identical to the zero DC offset curves in all panels of Fig. 3. The black dotted line in the ‘DC Component’ panel of (**c**) shows the threshold curve from the cathodal DC component (**b**) for comparison.

We quantified the contributions of the KHF and DC components of the signals to the production of conduction block for symmetric waveforms with amplitude- and frequency-dependent DC offsets of ± 4 μA DC per mA KHF per 1 kHz (Fig. 1c, subpanel c3) in a computational model of a 5.7 μm diameter fiber. To isolate the effects of the KHF and DC components, we filtered the waveforms to preserve either the KHF component only (high pass) or the DC offset component only (low pass) (Fig. 6a), and we found block threshold for each component separately.

Non-monotonic changes in block threshold with frequency reflected a transition from a purely KHF block regime at low frequencies, where the DC component of waveforms was small, to a block regime at high frequencies that was solely the result of the DC component as a consequence of the frequency- and amplitude-dependent increase in net DC offsets. The original waveforms resulted in non-monotonic block thresholds with frequency, and the KHF components of the original waveforms had thresholds that increased monotonically with frequency irrespective of the original waveform’s DC offset polarity (Fig. 6b,c). These results were identical to results for ± 4 μA DC per mA KHF per 1 kHz DC offset waveforms (i.e., the original waveforms) and for the 0 μA DC waveforms (i.e., KHF component only) shown in Fig. 3e,f. The DC offset components of the original waveforms had monotonically decreasing block thresholds regardless of DC offset polarity (Fig. 6b,c), reflecting the fact that the DC component of the original waveform had a larger magnitude at higher frequencies due to the DC offset being dependent on the original waveform’s KHF amplitude and frequency (Fig. 1c, subpanel c3). Therefore, at higher frequencies, the DC offset components extracted from the original waveform required a smaller pre-filtered KHF amplitude to reach DC block threshold. Block thresholds for the original waveforms approached the thresholds for the KHF-only components at lower frequencies and approached the thresholds for the DC offset components at higher frequencies, irrespective of DC offset polarity (Fig. 6b,c, Overlay), indicating that a transition from KHF to DC block underlays the non-monotonic threshold-frequency relationships of the original waveforms.

Cathodal DC components alone had lower block thresholds than anodal DC components alone (Fig. 6c, purple vs. black dotted lines), consistent with differences observed for symmetric waveforms at high frequencies with anodal versus cathodal DC offsets (Fig. 3f, − 4 vs. + 4 μA DC per mA KHF per 1 kHz lines). This polarity difference was due to anodal DC at the proximal contact augmenting incoming action potentials (Supplementary Video 1), as a result of sodium channel de-inactivation, allowing them to propagate through the distal cathode that otherwise could block action potentials when cathodal DC was at the proximal contact (Supplemental Video 2).

Our analysis further revealed that polarity-dependent differences in non-monotonic threshold-frequency relationships were due to polarity-dependent interactions between KHF and DC components during the transition from KHF to DC block regimes. For waveforms with anodal DC offsets, the transition was relatively smooth across frequencies, and block thresholds were always less than or equal to the KHF or DC components’ block thresholds. This result indicated a synergy between KHF and anodal DC (i.e., anodal DC at the proximal contact with cathodal DC at the distal contact) at all frequencies. In contrast, for waveforms with cathodal DC offsets, the transition was marked by an abrupt drop in thresholds after the ‘knee’ frequency (Fig. 6b, orange line, 29.4 vs. 38.5 kHz). Further, block thresholds leading up to this ‘knee’ frequency were greater than the KHF components’ block thresholds, but always less than or equal to the DC component’s block thresholds. This result indicated a reduced ability of KHF to block in the presence of cathodal DC offsets (i.e., before the ‘knee’) despite KHF always assisting the production of DC block (i.e., after the ‘knee’).

### Frequency-dependent charge imbalance blocked some smaller fibers at lower thresholds than larger fibers

**Figure 7:**
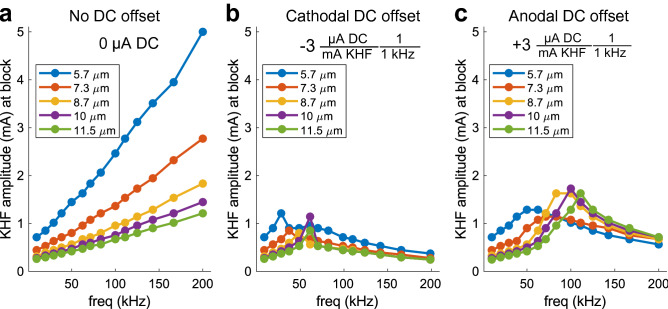
KHF block across modeled axons of multiple fiber diameters without (**a**) and with (**b**,**c**) amplitude- and frequency-dependent DC offsets. Each model axon was placed at the center of the rat tibial nerve FEM, and all axon lengths were 100 mm.

Using our computational model, we compared the frequency-dependent effects on block thresholds of symmetric rectangular waveforms with different DC offsets across fiber diameters (5.7, 7.3, 8.7, 10.0, 11.5 μm), extending the upper range of frequencies to observe frequency effects fully (111.1, 125, 142.6, 166.7, and 200 kHz). Block thresholds of KHF waveforms with no DC offset increased monotonically with frequency for all fiber diameters (Fig. 7a), while KHF waveforms with frequency- and amplitude-dependent charge imbalances produced non-monotonic threshold-frequency relationships for all fiber diameters (Fig. 7b,c). Further, block thresholds at any given frequency were inversely related to fiber diameter when no DC offsets were present (Fig. 7a), while non-monotonic frequency effects for both cathodal and anodal DC offsets resulted in instances where the order of block was reversed (Fig. 7b,c), such that smaller diameter fibers had lower block thresholds than larger diameter fibers. For cathodal DC offsets, such reversals occurred at specific frequencies and for specific fiber diameters (e.g., Fig. 7b, 10.0 μm vs. 5.7 μm at 62.5 kHz). For anodal DC offsets, reversals occurred starting at 71.4 kHz and were maintained across higher frequencies (Fig. 7c), resulting in reversal of block thresholds across all fiber diameters by 111.1 kHz.

### Interactions between KHF signal and DC offset modulated excitation and block regions

Our results showed that DC modulation of KHF block thresholds created non-monotonic relationships between block threshold and frequency when the DC offset was amplitude- and frequency-dependent. However, block threshold alone does not reflect the range of effects of KHF signals across amplitudes. Other responses, including transmission, excitation, and the extent of block across amplitudes (i.e., the block window) are highly relevant for in vivo application of block. Therefore, we further characterized the responses to KHF rectangular waveforms mixed with DC in computational models of 5.7 μm diameter myelinated fibers by analyzing the number of action potentials detected across amplitudes and frequencies of the KHF signals.

**Figure 8:**
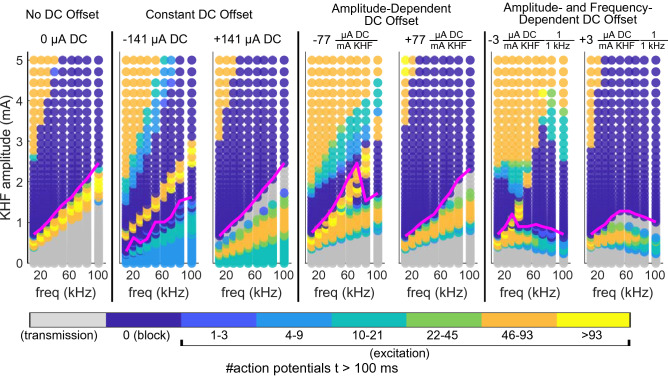
Representative examples of KHF amplitude and frequency effects on transmission, excitation, and block across a range of DC offset types for symmetric KHF waveforms from 10 to 100 kHz in computational models of 5.7 μm diameter myelinated fibers. Heatmaps show the number of action potentials that occurred between t = 100 and 250 ms at all KHF amplitudes, frequencies, and polarities across a representative subset of DC offset levels from Fig. 3. Action potential counts were binned and color-coded (colorbar). The type, amount, and polarity of DC offsets are labeled above each plot. The signs of the DC offsets denote the polarity delivered to the proximal contact (Fig. 2). DC offset types are labeled above each group of plots. KHF amplitudes were sampled from 0.05 to 5 mA in 6% increments. Gray transmission dots indicate the presence of exactly three action potentials spaced apart in time by 50 ms, corresponding to the number and timing of test pulses between 100 to 250 ms. KHF amplitude was half of the peak-to-peak KHF waveform amplitude. Magenta lines show block threshold curves from corresponding panels in Fig. 3.

Quantifying model responses across frequencies and amplitudes revealed that DC offsets caused gradual migration of KHF transmission, excitation, and block regions in ways that depended on the amount, polarity, and type of DC offsets. At low KHF amplitudes, waveforms with no DC offset (Fig. 1a) had no effect on action potentials produced by test pulses, i.e., transmission occurred (Fig. 8, 0 μA DC, gray dots). As the KHF amplitude was increased for a given frequency, the response progressed through tonic excitation by the KHF signal, conduction block, and then re-excitation (i.e., excitation by the KHF signal at amplitudes above the block threshold^42^). The range of amplitudes and frequencies that blocked axonal conduction formed a single contiguous region. Excitation, block, and re-excitation thresholds increased with frequency.

Anodal DC offsets of all three types (Fig. 1c) decreased the KHF amplitudes needed for KHF excitation, increased the KHF amplitudes needed for KHF re-excitation, and produced an additional transmission ‘region’ at KHF amplitudes just below block threshold (Fig. 8, + 141 μA DC, + 77 μA DC per mA KHF, + 3 μA DC per mA KHF per 1 kHz). Cathodal DC offsets of all three types had the opposite effect on KHF excitation and KHF re-excitation, and further produced an additional block ‘region’ and an additional excitation ‘region’ at KHF amplitudes below KHF excitation (Fig. 8, − 141 μA DC, − 77 μA DC per mA KHF, − 3 μA DC per mA KHF per 1 kHz). The additional transmission and block regions introduced by anodal and cathodal DC offsets, respectively, occurred at similar KHF amplitudes and frequencies, such that a given KHF signal could block action potentials coming from one direction but transmit action potentials coming from the other direction (see Supplemental Video 3 vs. Supplemental Video 4). The ‘knee’ frequency for − 3 μA DC per mA KHF per 1 kHz coincided with an abrupt transition from one block region to another. Together with the results from Fig. 6, which showed that the ‘knee’ represents a transition from a KHF to a DC block regime, these analyses indicate that the second block region introduced by cathodal DC offsets is a DC block region. Only the amplitude- and frequency-dependent DC offsets produced non-monotonic transmission, excitation, and block boundaries. These analyses demonstrate the complex effects that DC offset can have on transmission, block, and excitation in response to KHF signals.

## Discussion

Reversible block of nerve activity using KHF electrical signals has potential applications across a wide range of diseases with pathophysiological neural activity. Reported non-monotonic relationships between block amplitude and signal frequency provide an exciting possibility to develop fiber-selective nerve block approaches^17^, but these findings had to be reconciled with conflicting experimental evidence^18^. Using high-fidelity computational models and in vivo experiments, we quantified the effects of KHF signals with a range of charge imbalances on KHF nerve block to clarify the mechanisms of non-monotonic threshold-frequency relationships. Block thresholds could indeed change non-monotonically with frequency, and non-monotonicity could result in smaller fibers being blocked at lower thresholds than larger fibers. These non-monotonic effects were due to amplitude- and frequency-dependent charge imbalances and not to waveform asymmetry.

The effects of DC offset on KHF responses were complex and polarity-dependent. Polarity effects were particularly unexpected given our use of a geometrically symmetric bipolar cuff electrode. Nevertheless, the mechanism of these effects can be readily understood in terms of constructive or destructive interactions between depolarization resulting from the KHF and polarization by the DC anodal or cathodal offsets. The distal contact is particularly important to this understanding, as block can only be detected at the distal end of the axon if the distal contact blocks or if the proximal contact blocks in the absence of excitation at the distal contact. Low-amplitude DC anodal offsets at the proximal contact decreased KHF block thresholds because both the cathodal DC and the KHF signal at the distal contact drove membrane depolarization; low-amplitude cathodal DC at the proximal contact had the opposite effect because anodal DC at the distal contact counteracted KHF depolarization. Higher-amplitude DC of either polarity reduced block thresholds compared to pure KHF because, in those cases, block was primarily due to DC. However, anodal DC at the proximal contact had a weaker effect because the proximal anode caused sodium channel de-inactivation, which augmented incoming action potentials and enabled them to propagate through the distal cathode that would otherwise block (Supplemental Videos 3 and 4). This phenomenon underlies the regions of transmission that emerged between excitation and block (e.g., Fig. 8, + 141 μA DC), resulting in block of action potentials coming from one direction and transmission of action potentials coming from the opposite direction, and thus presenting the interesting possibility of unidirectional block with bipolar cuffs. Meanwhile, changes in KHF amplitude needed for re-excitation occurred because virtual DC cathodes (or virtual DC anodes) at the distal contact strengthened (or weakened) the depolarization at the virtual cathodes of the KHF signal, which are the source of KHF re-excitation^42^. The observed polarity effects on block thresholds were consistent with in vivo DC block measurements from a previous study that used both monopolar and bipolar cuffs^43^ and with prior modeling of monopolar electrodes^22^. There were no instances of anodal block in our computational models, and we also did not observe polarity effects in charge-balanced waveforms or waveforms with the lowest imbalances tested.

Our data confirm the predictions from prior modeling studies^21,22^ that reported non-monotonic block thresholds with frequency in charge-imbalanced asymmetric waveforms with a monopolar point source electrode in a homogeneous medium. However, we used more realistic preclinical computational models and validated our findings with in vivo experiments. Further, our use of DC offsets, asymmetric waveforms, and asymmetric charge-balanced waveforms revealed that asymmetry was neither necessary nor sufficient for non-monotonic block thresholds across frequencies, but rather that charge imbalances that scale with KHF amplitude and frequency are required to cause non-monotonicity. Indeed, asymmetry in the absence of charge imbalance caused monotonic frequency effects with the same thresholds as for charge-balanced symmetric waveforms.

Our findings clarify that non-monotonic frequency effects represent a transition from KHF block to DC block. This transition exhibited complex characteristics beyond block threshold effects, such as the shifting, broadening, and even splitting of excitation regions (Fig. 8). These results are relevant to approaches seeking to implement DC offsets into KHF waveforms^44,45^, as the alteration of excitation and block regions can reduce the available block window, making it harder to achieve and maintain nerve block. We did not confirm these excitation results in our in vivo experiments, as our experiments only characterized the minimum amplitudes required to block. Previous work showed that several characteristics of KHF excitation in models were reproduced in vivo^15^, although certain phenomena such as KHF re-excitation^42^ remain to be demonstrated.

Previous studies by Butera and colleagues with sea slugs, frog sciatic nerve, rat vagus nerve, and rat sciatic nerve reported that block thresholds as a function of frequency differed across fiber types within a nerve, such that slower fibers exhibited thresholds that changed non-monotonically from 5 to 50 kHz while faster fibers exhibited thresholds that increased monotonically^17,46,47^. These effects resulted in low-frequency block thresholds that were lower for faster fibers and high-frequency block thresholds that were lower for slower fibers, indicating potential for selective block of small fibers. Our results reveal that the non-monotonicity of block thresholds across frequencies is caused by frequency-dependent charge imbalance, suggesting that the effects observed in these prior studies may have occurred due to imperfect current sources. This explanation also reconciles the findings with a subsequent in vivo study showing only monotonic increases in block thresholds from 10 to 80 kHz across fiber conduction velocities in rat vagus nerve^18^. In contrast to the latter study, the former studies used a stimulator that was not designed for frequencies above 40 kHz, and use of higher frequencies—especially with higher loads—can distort waveforms and introduce amplitude- and frequency-dependent DC offsets (Supplemental Fig. 3). Since the former studies did not use a high pass filter circuit on the output of their stimulator^20^, the presence of inadvertent DC contamination remains a possibility. Monotonic increases in block threshold with frequency are expected with charge-balanced sinusoidal or rectangular KHF waveforms due to the low pass filtering property of the axonal membrane^19,48^. While frequency-dependent membrane capacitance reduces low pass filtering in computational models^49,50^, previous measurements showed only a factor of 2 reduction in capacitance between 10 and 100 kHz^51,52^, rather than the factor of > 10 reduction needed to reverse the low pass filtering and generate non-monotonic thresholds within this frequency range. Taken together with our results, the implication is that non-monotonic frequency dependent block thresholds arise from charge imbalances due to DC offsets or due to charge-imbalanced waveform asymmetries. Future studies of waveform effects across fiber types should explicitly quantify intentional charge imbalances or use a high pass filter circuit when imbalances are not intended^18,20^.

Our computational models showed that KHF waveforms with amplitude- and frequency-dependent charge imbalances enabled block of smaller fibers with lower amplitudes than larger fibers. In the light of advances in electrode materials that permit safe long-term DC nerve block^53,54^, our results suggest that controlled DC offsets are a feasible approach for fiber-selective conduction block through tuning the KHF frequency and relative amount of DC offsets. Therefore, our findings reinforce the use of frequency for fiber-selective block suggested by Butera and colleagues^17,46,47^ while elucidating the mechanism of action (i.e., DC offsets mixed with KHF). An important distinction is that those studies reported non-monotonic behavior only in small fibers but not in large fibers, although perhaps at higher frequencies they would have observed non-monotonic behavior across all fiber sizes, as reported here. As a consequence of all fiber diameters exhibiting non-monotonic frequency effects, the relationship between frequency and blocked fibers is not straightforward, and subsequent studies using single-unit recordings may be needed to characterize the degree of selectivity possible with this technique. Further, our simulations were exclusively of myelinated fibers, and specific threshold-frequency relationships in response to KHF with DC offsets may differ in unmyelinated axons (although monotonic frequency effects in the absence of DC offsets are still expected, per the membrane filtering discussed in the previous paragraph).

Cumulative application of DC nerve block can damage nerves due to the production of reactive species resulting from electrode polarization^23^. We did not systematically quantify the degree of DC offset that leads to nerve damage or electrode degradation in vivo, but we mitigated the potential acute effects of DC by applying all waveforms at “cathodal” and “anodal” polarities back-to-back to maintain net charge balance across trials. All nerves in our study continued to conduct action potentials throughout the entire experiment. The DC offsets required for non-monotonic block thresholds with increasing frequency are expected to be smaller than pure DC block given that the DC and KHF components both contribute to nerve block. Nevertheless, the potential for nerve damage currently limits the ability to apply KHF with DC offsets clinically. Clinical translation will require electrode materials with higher charge storage capacity^53,54^, as well as a clear understanding of the non-damaging limits^55^ for KHF with DC offsets within chronic preclinical environments.

In conclusion, we found that block threshold changed non-monotonically with frequency when DC offsets scaled with KHF amplitude and frequency. Charge imbalances could be achieved with either DC offset or with waveform asymmetry. Non-monotonic frequency effects resulted in instances where smaller fibers were blocked at lower thresholds than larger fibers. However, a clear relationship between frequency, DC offsets, and fibers blocked remains to be established experimentally. DC offsets produced complex patterns of excitation, transmission, and block as frequency and amplitude were varied, and this complexity may pose an obstacle to clinical translation efforts. Finally, the use of DC offsets with KHF signals for chronic applications currently has the potential for nerve damage, and there is a need for new materials and studies to enable non-damaging application of DC offsets with KHF.

## Supplementary Information

Supplementary Information 1.

Supplementary Video 1.

Supplementary Video 2.

Supplementary Video 3.

Supplementary Video 4.

## Data Availability

Block thresholds from all waveforms and frequencies from computational models and in vivo experiments were uploaded to Duke University’s Research Data Repository. Data are persistently accessible at 10.7924/r4pn94v5h.
