# Supplementary material for: Non-monotonic kilohertz frequency neural block thresholds arise from amplitude- and frequency-dependent charge imbalance
Source: Sci Rep. 2021 Mar 3;11:5077. doi: 10.1038/s41598-021-84503-3 (PMC7930193; doi:10.1038/s41598-021-84503-3)
Supplement: Supplementary file 1 — Supplementary Information 1. [file 41598_2021_84503_MOESM1_ESM.pdf]

Non-monotonic kilohertz frequency neural block thresholds arise from amplitude- and frequency-dependent charge imbalance

Edgar Peña[1], Nicole A. Pelot[1], Warren M. Grill[1,2,3,4]

[1] Department of Biomedical Engineering
Duke University
Room 1427, Fitzpatrick CIEMAS
101 Science Drive
Campus Box 90281
Durham, NC 27708

[2] Duke University, Department of Electrical and Computer Engineering, Durham, NC, USA
[3] Duke University School of Medicine, Department of Neurobiology, Durham, NC, USA
[4] Duke University School of Medicine, Department of Neurosurgery, Durham, NC, USA

Corresponding author: warren.grill@duke.edu

Supplemental Figures

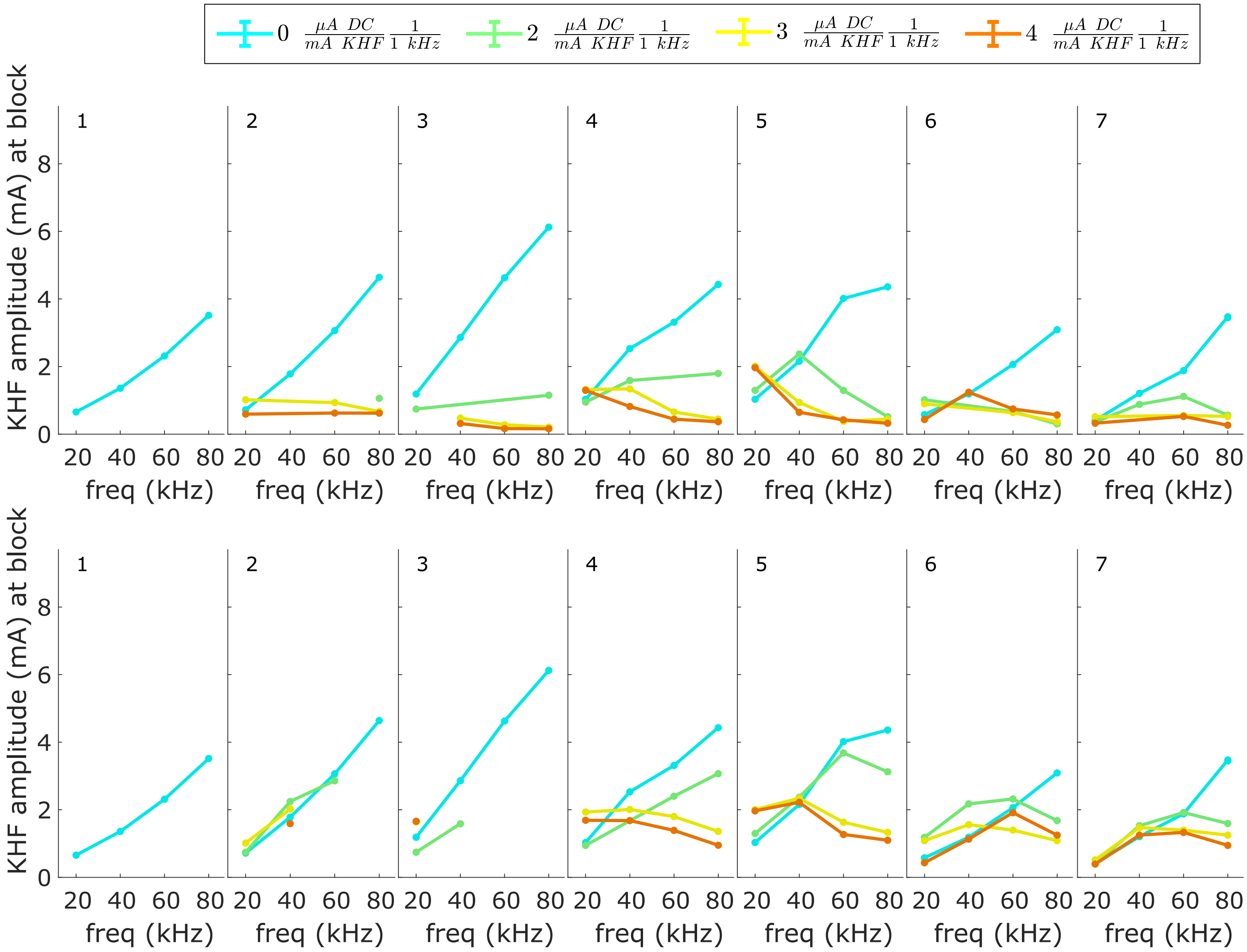

Supplemental Figure 1: Individual data across all seven nerves in symmetric waveforms for cathodal (top) and anodal (bottom) DC offsets.

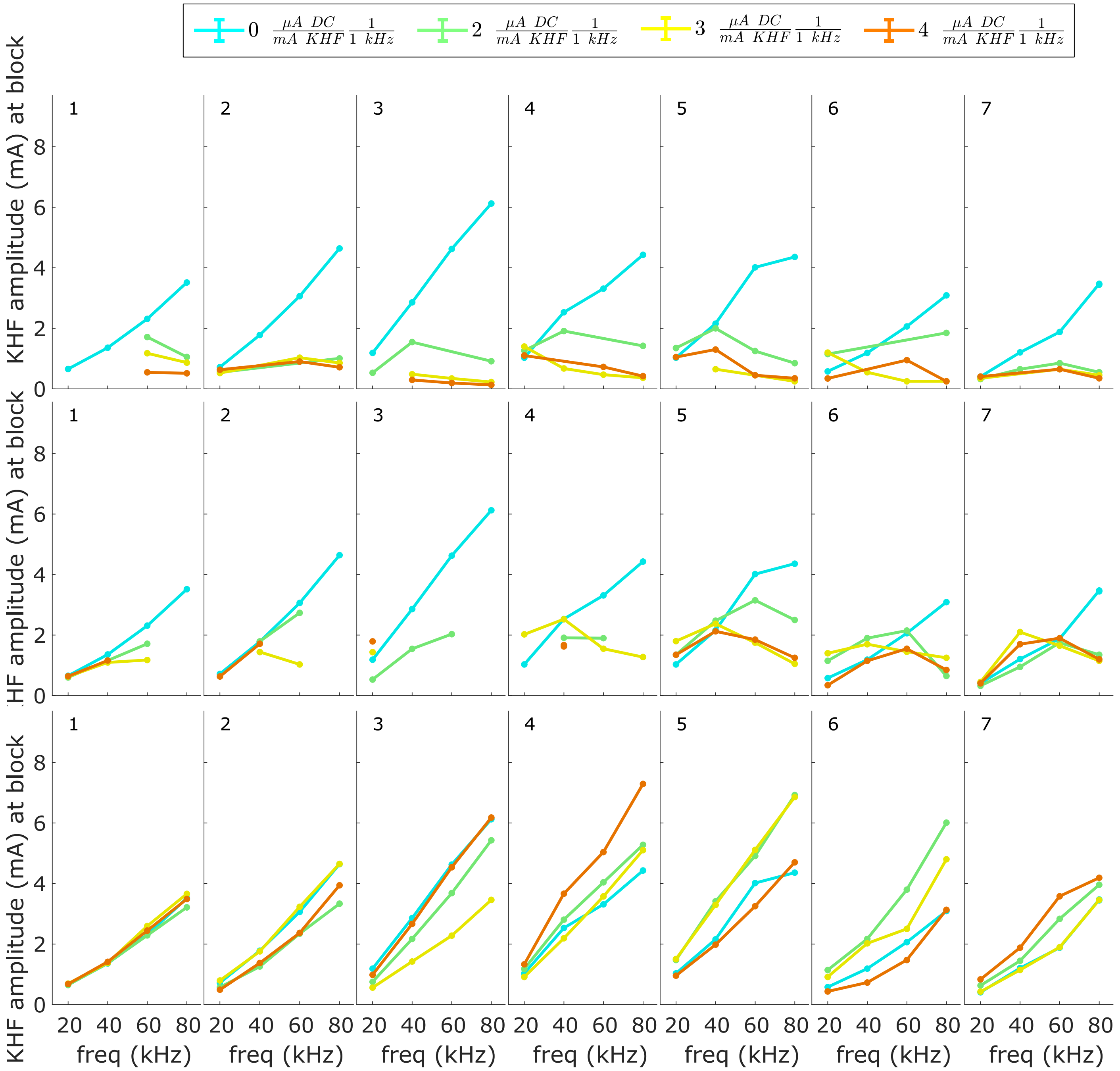

Supplemental Figure 2: Individual data across all seven nerves in asymmetric waveforms for longer cathodal phase charge-imbalanced (top), longer anodal phase charge-imbalanced (middle), and charge-balanced asymmetric.

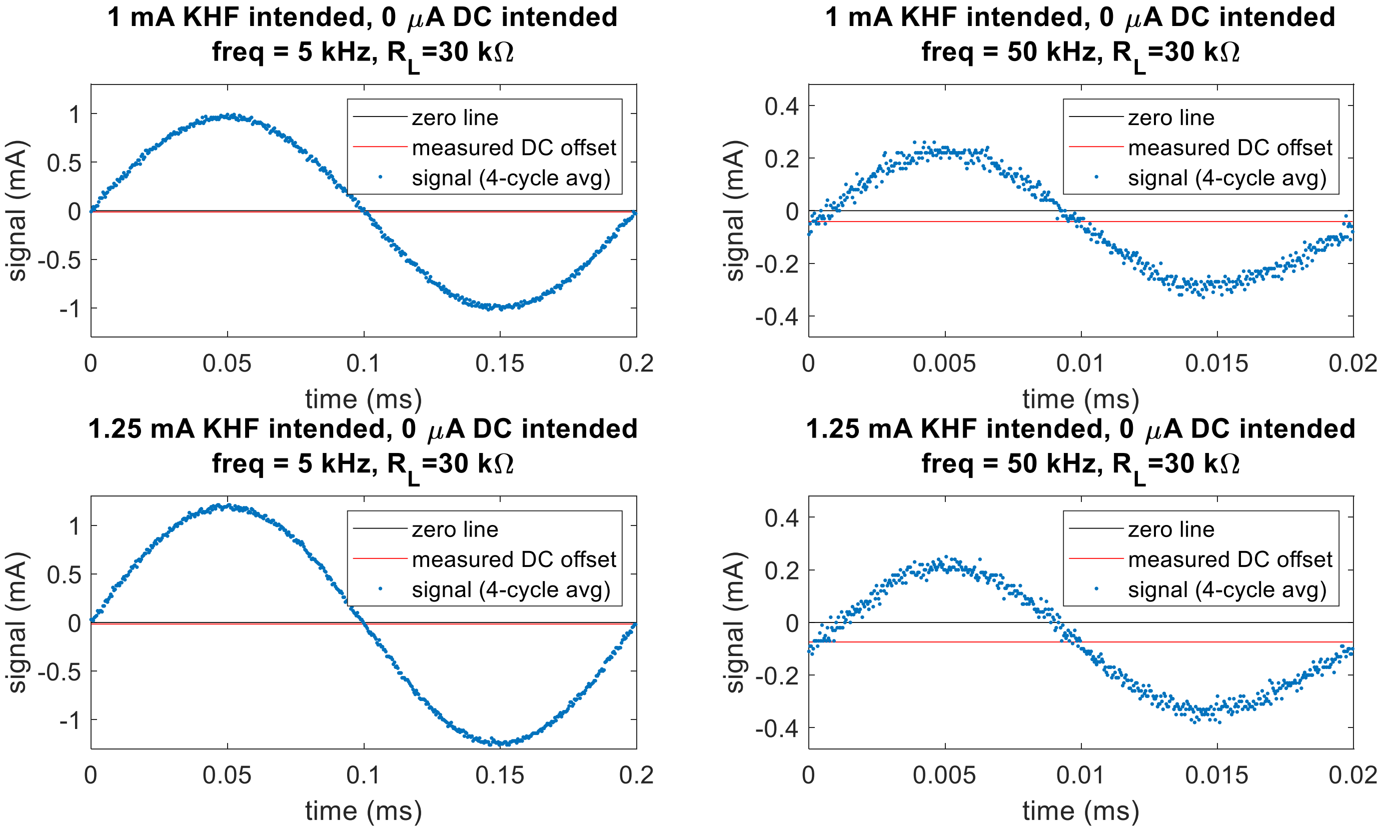

Supplemental Figure 3: Oscilloscope recordings (Tektronix tbs1032b) of kilohertz signals generated using the stimulator and load size reported in (Joseph and Butera 2009) (A-M Systems 2200, 30 kΩ resistive load) at two different amplitudes (1 mA & 1.25 mA) and two different frequencies (5 kHz & 50 kHz). DC offsets at 5 kHz were small (~-13 µA DC per actual mA of KHF at 1 mA & 1.25 mA intended KHF), but DC offsets at 50 kHz were large (~-164 and ~-274 µA DC per actual mA of KHF at 1 mA & 1.25 mA intended KHF). The change in DC offsets per intended mA KHF from 5 kHz to 50 kHz was comparable to the DC offsets we showed to be necessary for non-monotonic thresholds (~-3.3 and ~-5.8 µA DC per actual mA KHF per kHz). The stimulator was calibrated by adjusting DC offset screw to output <2 µA when the input was 0 V. Plots show average of four recorded cycles. DC offset was estimated from area under the curve of the average of the four recorded cycles using trapz in MATLAB R2018a.
